# A human gut bacterial genome and culture collection for improved metagenomic analyses

**DOI:** 10.1038/s41587-018-0009-7

**Published:** 2019-02-04

**Authors:** Samuel C. Forster, Nitin Kumar, Blessing O. Anonye, Alexandre Almeida, Elisa Viciani, Mark D. Stares, Matthew Dunn, Tapoka T. Mkandawire, Ana Zhu, Yan Shao, Lindsay J. Pike, Thomas Louie, Hilary P. Browne, Alex L. Mitchell, B. Anne Neville, Robert D. Finn, Trevor D. Lawley

**Affiliations:** 1 Host-Microbiota Interactions Laboratory, Wellcome Sanger Institute, Wellcome Genome Campus, Hinxton, UK; 2 Centre for Innate Immunity and Infectious Diseases, Hudson Institute of Medical Research, Clayton, Victoria Australia; 3 Department of Molecular and Translational Sciences, Monash University, Clayton, Victoria Australia; 4 European Molecular Biology Laboratory, European Bioinformatics Institute, Wellcome Genome Campus, Hinxton, UK; 5 Bacterial Genomics and Evolution Laboratory, Wellcome Sanger Institute, Wellcome Genome Campus, Hinxton, UK; 6 Department of Microbiology and Infectious Diseases, University of Calgary, Calgary, Alberta Canada; 7 Present Address: Microbiology and Infection Unit, Division of Biomedical Sciences, Warwick Medical School, University of Warwick, Coventry, UK

**Keywords:** Bacterial genomics, Metagenomics, Microbiome

## Abstract

Understanding gut microbiome functions requires cultivated bacteria for experimental validation and reference bacterial genome sequences to interpret metagenome datasets and guide functional analyses. We present the Human Gastrointestinal Bacteria Culture Collection (HBC), a comprehensive set of 737 whole-genome-sequenced bacterial isolates, representing 273 species (105 novel species) from 31 families found in the human gastrointestinal microbiota. The HBC increases the number of bacterial genomes derived from human gastrointestinal microbiota by 37%. The resulting global Human Gastrointestinal Bacteria Genome Collection (HGG) classifies 83% of genera by abundance across 13,490 shotgun-sequenced metagenomic samples, improves taxonomic classification by 61% compared to the Human Microbiome Project (HMP) genome collection and achieves subspecies-level classification for almost 50% of sequences. The improved resource of gastrointestinal bacterial reference sequences circumvents dependence on de novo assembly of metagenomes and enables accurate and cost-effective shotgun metagenomic analyses of human gastrointestinal microbiota.

## Abstract (web summary)

A large bacterial strain collection and genome sequences will boost gut microbiome research.

## Main

The human gastrointestinal tract harbors a diverse and dynamic microbial community that directly impacts human biology and health^1–3^. This complex ecosystem is dominated by bacteria, but also includes viruses, archaea, fungi and other eukaryotes. Metagenomic sequencing is the main method used to study GI tract microbiomes and other microbiomes in both natural and built environments^1,2,4^. Amplicon sequencing, targeting the 16 S ribosomal RNA (rRNA) gene, enables characterization of taxonomic level bacterial and archaeal compositions and can detect structural changes in microbial communities. However, biologically relevant phenotypic differences exist even between highly related bacterial strains of the same species^5^, and these strain differences cannot typically be distinguished by amplicon sequencing. Using shotgun metagenomic sequencing, it is feasible to assess the entire genomic content of any microbiome and achieve precise taxonomic classification and accurate functional assignments, but only if metagenome sequences can be interpreted to reveal all the species and strains present^1^.

Computational approaches can be applied to extract species- and even subspecies-level information from metagenomic samples^1–3,6–10^; however, these approaches are fundamentally constrained by their requirement for deep sequence coverage and the inability to differentiate between closely related bacterial taxa^11^. Furthermore, genomes derived from de novo metagenomic assemblies may be incomplete or may represent chimeric species populations, unlike high-quality reference genomes generated from pure cultures^12^. These factors limit the accuracy of high-resolution taxonomic classification and functional analysis using metagenome-derived genomes. There is evidence that many people harbor multiple strains of the same bacterial species in their gastrointestinal microbiota^6^, which means there is a pressing need to improve the precision and accuracy of metagenomic analyses to enable the functional validation that is required to develop microbiome-based therapeutics^13,14^.

Comprehensive collections of reference-quality bacterial genomes enable accurate, reference-based metagenomic analysis (RBMA) and achieve species-, subspecies- and strain-level taxonomic classification of the bacterial composition of a microbiome. Substantial effort has been devoted to assembling bacterial reference genomes from different environments^15^ including the Human Microbiome Project (HMP), which has sequenced bacterial isolates from 18 human body sites^16^; however, due to the diversity between individuals and previous limits in culturing methods, the majority of species still remain to be isolated, archived and genome sequenced. With recent advances in bacterial culturing methods, it is now possible to grow and purify most bacteria from the human GI tract in the laboratory^17–20^.

In addition to genome sequences, access to archived bacterial isolates for functional experiments facilitates the transition from sequence-based, correlative studies to causative phenotypic validation of predicted bacterial function^13^. We report compilation and sequencing of the Human Gastrointestinal Bacteria Culture Collection (HBC), which contains isolates from the human GI tract and should enable accurate metagenomic analyses without a requirement for de novo assembly or ultra-deep sequencing and experimental validation.

## Results

### Assembly of a gastrointestinal bacteria culture collection

To assemble a comprehensive collection of bacterial isolates from the human GI tract, we cultured and purified bacterial isolates from fecal samples of 20 adults based in the United Kingdom (*n* = 8) and North America (*n* = 12). In total, we picked more than 10,000 bacterial isolates that were then taxonomically classified using 16 S rRNA gene sequencing. Combined with 234 GI tract isolates that we previously reported^17^, 737 purified and archived isolates are now included in the HBC. This collection represents 273 species (105 novel species) from 31 families in the phyla Actinobacteria (53 genomes; 16 species), Bacteroidetes (143 genomes; 40 species), Firmicutes (496 genomes; 203 species) and Proteobacteria (45 genomes; 14 species) (Supplementary Table 1). A genome sequence is available for each isolate in the HBC.

We combined our HBC genomes with 617 publicly available, high-quality human gastrointestinal-associated bacterial genomes available through the National Center for Biotechnology Information (NCBI) genome database to generate the Human Gastrointestinal Microbiota Genome Collection (HGG; Supplementary Table 1). Notably, 53% of species represented in the HGG genomes are archived in the HBC. Many of the remaining species currently absent from the HBC but present in the HGG include members of the Fusobacteria, Proteobacteria and Synergistetes, which are typically absent from healthy individuals in the developed world. This suggests further targeted culturing is required from a more diverse cohort of healthy donors and those affected with disease to exhaustively archive the bacterial component of the human GI tract microbiota.

**Fig. 1:**
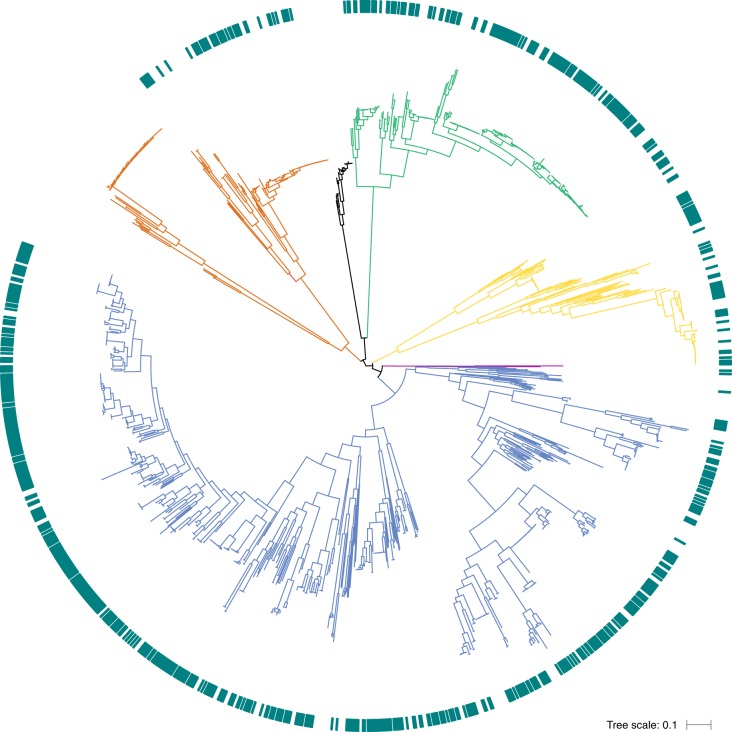
Phylogenetic diversity of the human gastrointestinal microbiota genome collection. Maximum-likelihood tree generated using the 40 universal core genes from the 737 HBC genomes (green outer circle) and the 617 high-quality public genomes derived from human gastrointestinal tract samples, which together make up the HGG. Branch color distinguishes bacterial phyla belonging to Actinobacteria (gold; *n* = 129 genomes), Bacteroidetes (green; *n* = 231 genomes), Firmicutes (blue; *n* = 772 genomes), Fusobacteria (black; *n* = 26 genomes), Synergistetes (pink; *n* = 2 genomes) and Proteobacteria (orange; *n* = 194 genomes) shown.

In total, the 1,354 genomes in the HGG represent 530 species from 57 families within the phyla Actinobacteria (129 genomes; 55 species), Bacteroidetes (231 genomes; 69 species), Firmicutes (772 genomes; 339 species), Fusobacteria (26 genomes; 9 species), Proteobacteria (194 genomes; 56 species) and Synergistetes (2 genomes; 2 species) (Supplementary Fig. 1). To understand the phylogenetic relationship between these taxa, we extracted 40 universal core genes^21^ from each genome and performed phylogenetic analysis (Fig. 1; Supplementary Fig. 2). Overall, the maximum phylogenetic diversity was observed in Firmicutes, particularly the classes Clostridia, Erysipelotrichia and Negativicutes; however, a broad range of species and phylogenetic group are represented across all phyla (Fig. 1; Supplementary Fig. 2).

### HGG improves gastrointestinal metagenome analyses

**Fig. 2:**
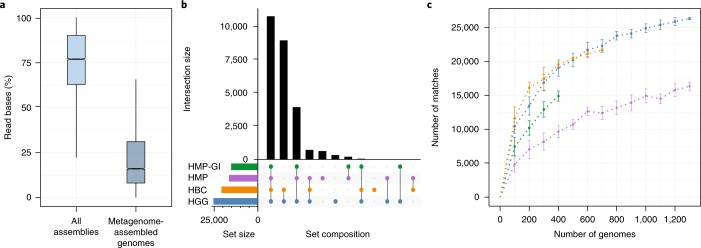
Comparison of high-quality reference genomes from de novo assembly and HGG. **a**, Read base usage as a percentage of total read bases present within the metagenomics samples (*n* = 13,490) that could be mapped to their respective de novo assembled contigs (min., 22.23; Q1: 62.87; median, 76.89; Q3, 89.99; max., 99.98) and metagenome-assembled genomes (MAGs; min., 0.16; Q1, 8.17; median, 16.09; Q3, 31.16; max., 65.64). **b**, Total number of classified bins using HGG (blue), genomes derived from the HBC collection alone (HBC; orange), the HMP (purple) and gastrointestinal derived isolates from the HMP (HMP-GI; green). **c**, Total number of 39,913 MAGs classified using subsampled sets of genomes from the HGG (blue), HBC (orange), HMP (purple) and HMP-GI (green). Error bars show mean and s.d. (*n* = 100 bootstraps).

In the absence of reference genomes, state-of-the-art analysis of metagenomic sequencing is dependent on de novo assembly of raw reads, followed by contig binning to generate metagenome-assembled genome sequences (MAGs). To compare the efficiency of taxonomic classification of de novo assembly and binning to RBMA analysis, we considered 13,490 publicly accessible (Supplementary Table 2) shotgun metagenomes from feces, with sufficient read coverage to perform de novo assembly. De novo assembly and contig binning identified 11,892 samples (88.2%) that were of sufficient quality to produce contigs greater than or equal to 2,000 base pairs (bp) in length. A total of 39,913 bins with >90% completeness and <5% contamination, referred to hereafter as MAGs, were obtained from 9,548 assemblies (Supplementary Table 3). Of these MAGs, 81% had at least 15 tRNAs, further emphasizing their high level of completeness; however, only 16.1% (interquartile range, IQR = 31.2%–8.2%) of read bases contributed to these MAGs (Fig. 2a).

To evaluate how the HBC genome collection alone and the complete HGG collection compares to the existing HMP genomes, we next considered which of the MAGs could be identified using each collection as a reference database. The HGG was able to identify 25,085 MAGs compared to 20,772 with the genomes corresponding to just the HBC. Meanwhile, 16,476 MAGs were identified with the HMP collection from all 18 body sites, and 15,156 MAGs were found when including only HMP isolates from the gastrointestinal body sites (HMP-GI). This represents a 52.3% improvement when using the HGG collection as a reference compared to the complete HMP (Fig. 2b). As the HGG collection is considerably larger than the HBC, HMP and HMP-GI genome collections, we next performed bootstrapped subsampling of each genome database and compared the selected genomes to the previously identified MAGs by average nucleotide identity (ANI > 95%). Considering subsamples of 400 genomes, the last data point available for the HMP-GI, the HGG achieves 19,545 matches and the HBC genome collection 19,036 matches compared to 14,906 matches with the HMP-GI and 9,655 with the full HMP (Fig. 2c). Classification is hindered in the full HMP, as it includes genomes from nongastrointestinal species. Notably, the greater matching achieved using the HGG and HBC genomes suggests more representative phylogenetic diversity is also present within these datasets. Thus, our analysis demonstrates a 61.1% increase in classification potential with the HGG compared to the existing genomes.

### Phylogeny-based estimate of genome coverage in metagenomes

**Fig. 3:**
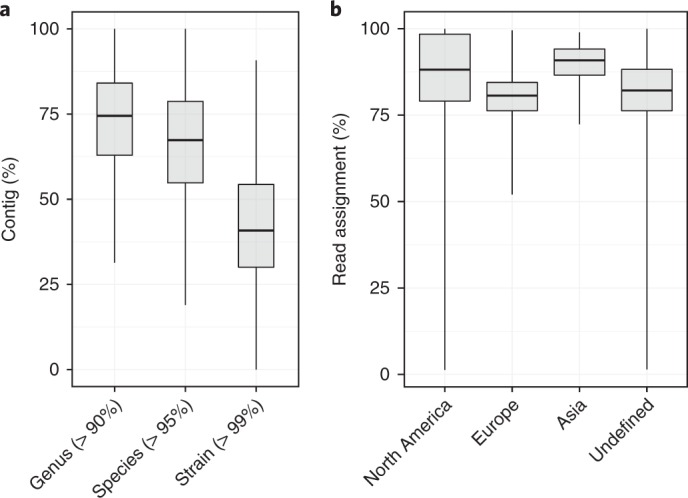
Classification efficiency using the HGG. **a**, Contig assignment from 13,490 metagenomic samples at genus (90%; min., 31.35; Q1, 62.92; median, 74.48; Q3, 84.10; max., 100.0), species (95%; min., 18.94; Q1, 54.80; median, 67.35; Q3, 78.73; max., 100.0) and strain (99%; min., 0.0; Q1, 30.03; median, 40.82; Q3, 54.35; max., 90.77) identity compared to the HGG. **b**, Classification of metagenomic sequenced samples from North America (*n* = 2,064; min., 1.31; Q1, 79.07; median, 88.16; Q3, 98.42; max., 99.97), Europe (*n* = 1,431; min., 52.07; Q1, 76.28; median, 80.66; Q3, 84.47; max., 99.52), Asia (*n* = 191; min., 72.37; Q1, 86.56; median, 90.84; Q3, 94.13; max., 98.93) and the other undefined locations (*n* = 9,804; min., 1.45; Q1, 76.28; median, 82.14; Q3, 88.25; max., 99.94).

Although it is possible to generate MAGs using de novo assembly and binning approaches, this method remains unable to assign 83.9% of reads considered within the 13,490 shotgun metagenomic sequenced samples analyzed in this study. To address this limitation, we next compared all de novo assembled contigs with the HGG to determine the ability to classify a larger proportion of the input data. Applying this method, we were able to map 74.5% (IQR = 84.1%–62.9%) of contigs at a level approximately equivalent to genus (90% cutoff), whereas we could assign 67.3% (IQR = 78.7%–54.8%) at the species level (95% cutoff; Fig. 3a). Remarkably, 40.8% (54.3% – 30.0%) could be classified below species level (99% cutoff) despite not including any isolates cultured from any of these samples in the HGG (Fig. 3a).

Given the improvements in classification that the HGG provides, we next adopted a lowest common ancestor RBMA to determine overall taxonomic classification efficiency across the same dataset. Compared to de novo metagenomic assembly and binning approaches, RBMA is more resilient to low sample coverage because it requires shallower sequencing depth to confidently assign a sequence to a reference genome. With these datasets, RBMA of large-scale shotgun metagenomic datasets required a median processing time of 7.3 min for each sample compared to the 12.19 h required for an equivalent de novo assembly. This substantial reduction in required computational performance provides a means to process more samples and overcome the limitations in statistical power that hinders many metagenomic studies.

Horizontal gene transfer of mobile elements in bacterial populations and communities can limit our ability to identify the true species composition because of incorrect read assignment. To address confounding factors associated with horizontal gene transfer and provide a more precise estimate of taxonomic coverage, we also generated a comprehensive list of mobile elements, insertion sequences and plasmids found within the European Nucleotide Archive (ENA)^22^. Combined with the mobile elements predicted in the HGG, this represents a comprehensive database of known mobile elements found within the human gastrointestinal microbiota. The database includes 2,803 plasmids and 489 transposons and insertion sequences that were masked within the genomes and filtered from metagenomic reads before lowest common ancestor classification to maximize the phylogenetic signal (Supplementary Table 4). When we applied the lowest common ancestor RBMA with the mobile element filtered HGG, classification of the raw reads achieved an average taxonomic assignment of 82.9% at the genus level and 78.7% at species levels. Taken together, these analyses reveal that high-resolution classification of the majority of metagenomic reads derived from the human gastrointestinal microbiota can be achieved using the HGG even when considering samples across diverse geographic populations (Fig. 3b).

### Bacterial diversity in the human gastrointestinal tract

We next sought to understand which species were most prevalent within the human gastrointestinal microbiota using the HGG. To do this, we interrogated all of the 13,940 high-quality shotgun metagenomic samples derived from human feces (Supplementary Table 2). Though this analysis may be impacted by variation in fecal sample storage conditions and DNA extraction methods^23^, we reasoned that those species that are highly prevalent across samples from many individuals are likely to play an important role in human biology and should be the focus of further investigation. Considering only species that are present at a level greater than 0.01% within any sample, we identified 165 species present across more than two unrelated samples (Supplementary Table 5). This group of dominant species included Bacteroidetes (*n* = 41), Firmicutes (*n* = 82), Proteobacteria (*n* = 27) and Actinobacteria (*n* = 15). Given the background prevalence of each phylum, this represents a significant overrepresentation of species from Bacteroidetes (*P* < 0.05) and a significant underrepresentation of species from Firmicutes (*P* < 0.01).

**Fig. 4:**
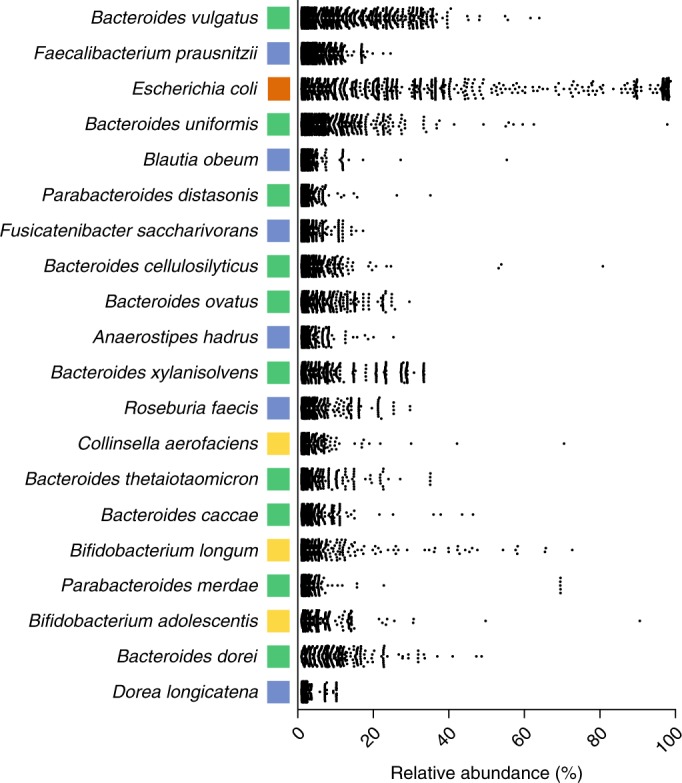
Dominant bacterial species within the human gastrointestinal microbiota. Dominant species, ordered by prevalence, found within the 13,490 human gastrointestinal metagenomic samples and their relative abundance within each sample. Color denotes Bacteroidetes (green), Firmicutes (blue), Proteobacteria (orange), Actinobacteria (gold).

Considering all species that were detected above background levels, the majority of dominant species remain as members of the Bacteroidetes. In total, 8 of the top 20 prevalent species were members of the *Bacteroides* genus (*Bacteroides vulgatus*, *Bacteroides uniformis*, *Bacteroides cellulosilyticus*, *Bacteroides ovatus*, *Bacteroides xylanisolvens*, *Bacteroides thetaiotaomicron*, *Bacteroides caccae* and *Bacteroides dorei*). When corrected for the number of species within each phylogenetic group, the Bacteroidetes generally, and the *Bacteroides* and *Parabacteroides* genera (*Parabacteroides distasonis* and *Parabacteroides merdae*) more specifically, are significantly overrepresented (*P* < 0.001; Fig. 4). Despite three being over 346 species within the Firmicutes, there are only 6 distantly related Firmicute species that were highly represented across many individuals (*Fecalibacterium prausnitzii*, *Blautia obeum*, *Fusicatenibacter saccharivorans*, *Anaerostipes hadrus*, *Roseburia faecis* and *Dorea longicatena*; Fig. 4). Overall, all detected genera within the Firmicutes phylum were statistically underrepresented in their occurrence. Similarly, the only member of the Proteobacteria that was highly prevalent across samples was *Escherichia coli*, with the majority of Proteobacteria not detected within the samples. Interestingly, no members of the Fusobacteria or Synergistetes were found to be prevalent at the level of detection considered, suggesting that they are found only during certain conditions or stages of life that were not included in this analysis.

These data suggest a potential key role for specific members of the Bacteroides within the human gastrointestinal tract. In contrast, the significantly greater diversity observed in Firmicutes, the other dominant phyla, suggests a highly variable, potentially functionally redundant group consistent with previous reports of dynamic spore-mediated transmission and turnover for many taxa within this group^17,24^. Although laboratory-based phenotypic analysis examining many of the key species that were identified through this study remains limited, this can now be addressed through access to the isolates archived in the HBC.

Given the diverse array of novel genomes contained within the HGG, we next sought to understand the prevalence of these species across the community. Importantly, the availability of these genomes allows us to reliably assess the prevalence of these species in metagenome samples for the first time. In total, 106 of the 173 novel genomes (60.9%) are found at greater than 0.001% abundance in at least one sample within the 13,490 public metagenome samples available. Notably, almost half (87; 48.6%) were found in >100 samples, but less than one quarter (39; 21.8%) were found in >1,000 samples. Interestingly three novel species all within the Clostridiales were found in almost half the samples analyzed. Two novel Lachnospiraceae were respectively found in 7,797 (55.9%) and 7,074 (50.7%) samples, and a new Ruminococcaceae species was found in 6,777 (48.6%) samples. Collectively, these data suggest many of the novel species and genomes identified through this work occur frequently within the human population and potentially represent integral parts of the human gastrointestinal microbiota that warrant further investigation.

### Functions of human gastrointestinal bacteria

This extended collection of genome sequenced bacterial isolates enables high-resolution functional and taxonomic analysis. We first performed a clusters of orthologous group of protein (COG) annotation^25^ on the protein sequences to identify those features prevalent within the HGG bacteria. This analysis identified 4,696 distinct orthologous groups represented in at least one isolate. As expected, bacterial housekeeping functions, including ribosomal protein function, amino acid synthesis and other translation-associated functions, dominate the 30 functions found in all bacteria within the collection (Supplementary Table 6).

**Fig. 5:**
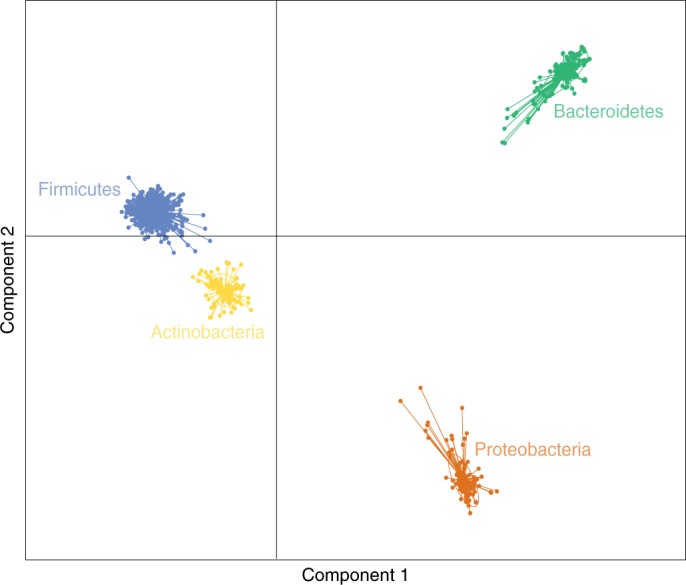
Bacterial functions in the human gastrointestinal tract. DAPC analysis of functional categories shows a clear separation of functions associated with each dominant phylum (Bacteroidetes (green; *n* = 231 genomes), Firmicutes (blue; 772 genomes), Proteobacteria (orange; *n* = 194 genomes), Actinobacteria (gold; *n* = 129 genomes)) within the HGG collection.

To understand differences in the functional role performed by members of the four major bacterial phyla of the gastrointestinal microbiota (Bacteroidetes, Firmicutes, Actinobacteria and Proteobacteria), we compared 4,696 orthologous groups identified with COG analysis using discriminant analysis of principle components (DAPC). This comparison demonstrates clear functional differences between key phyla of the human gastrointestinal microbiota (Fig. 5). Next, we undertook an enrichment analysis to identify those functions overrepresented in each phylum relative to all functions present within the HGG. This analysis identified 8, 122, 152 and 389 statistically enriched functions (*q* < 0.001) in Actinobacteria, Bacteroidetes, Firmicutes and Proteobacteria, respectively (Supplementary Table 7). Enriched functions within the Actinobacteria were limited, but those identified were primarily associated with lipid (*q* < 1.99 × 10^−83^) and carbohydrate metabolism (*q* < 7.57 × 10^−77^). Equivalent analysis of the Bacteroidetes specific functions identified many key functions, including iron (*q* < 1.18 × 10^−114^) and sulfur transporter functions (*q* < 6.82 × 10^−97^) and specific sodium-transporting NADH ubiquinone oxidoreductases (*q* < 3.47 × 10^−124^). Firmicutes were dominated by uncharacterized functions; however, spore formation (*q* < 3.48 × 10^−123^) and thiamine (*q* < 2.76 × 10^−101^) and riboflavin (*q* < 7.04 × 10^−101^) transport were all highly enriched. Finally, Proteobacteria were dominated by fructose bisphosphatase (*q* < 4.50 × 10^−140^), glucokinases (*q* < 4.55 × 10^−125^) and regulators of iron cluster formation (*q* < 9.20 × 10^−98^). These results demonstrate the distinct differences in the unique functions provided by the key phyla of the human gastrointestinal microbiota; however, the prevalence of uncharacterized functions further demonstrates the need for better genome annotation and functional genomics to understand these bacteria.

The HGG collection contains genomes from 173 species not previously isolated from the human gastrointestinal tract. This includes genomes from the 105 novel species within the HBC and genomes from 68 known species in which genome-sequenced isolates from the human gastrointestinal tract did not previously exist (Supplementary Table 1). To understand what functions were found within these 173 species but were absent within the previously reported genome-sequenced species, we performed functional analysis. In total, 45 newly described functions, of which 41 were found in the Firmicutes, were identified. Though these functions were dominated by uncharacterized proteins, novel functions included those associated with tetrahydromethanopterin *S*-methyltransferase (present in five species), preprotein translocase (also present in five species) and formaldehyde-activating enzyme necessary for methanogenesis (found in four of the previously uncharacterized Firmicutes). In addition, 83.2% of these newly sequenced isolates and 85.8% of the novel species are predicted to form spores on the basis of previously defined genomic signatures^17^.

Finally, we sought to understand which functions were predicted to occur in newly genome-sequenced members of a particular phyla but were absent in all existing genomes of that phyla. This analysis identified type III, IV and VI secretion system components in Bacteroidetes that were not found in any of the previously sequenced gastrointestinal Bacteroidetes but were recognized within the existing Proteobacteria and Firmicutes genomes. Equally, ABC transporter functions found in existing genomes from Proteobacteria were identified within the newly sequenced gastrointestinal Firmicutes but not within any of the previously sequenced isolates. This suggests further functional overlap that may exist between specific members of phyla with potentially important, redundant roles in microbial community dynamics and host–microbiota interactions.

## Discussion

We present a gastrointestinal bacteria genome and culture collection that substantially increases the proportion of species found in metagenomics samples from the developed world. The YCFA medium used achieves bacterial growth at levels broadly representative of the original sample, so it will therefore be necessary to combine YCFA with selective culturing techniques to target specific bacterial phenotypes; for example, antibiotic resistance, sporulation, carbohydrate utilization and isolation of rare bacterial species present in individual fecal samples. Shotgun metagenomic sequencing has not been performed for many of the world’s population, so it is not currently possible to accurately assess the proportion of cultured bacteria across the entirety of the human population. We proposed that an expanded, coordinated, global culturing exercise with particular focus on samples and bacterial isolates from the developing world and more diverse communities across the developed world is needed. Collection and storage of metadata associated with these metagenomic samples is also essential: despite efforts to develop standards^26,27^, many genome and metagenomic sequences deposited in the public sequence collections have incorrect, inconsistent, missing or limited metadata that fundamentally limits their use.

In addition to improved species classification, access to comprehensive genome sequenced isolates fundamentally alters the methods, resolution and accuracy of functional analysis. Genome-sequenced isolates enable functional capacity to be inferred from the genetic repertoire of the reference genomes. This eliminates the need to perform ultra-deep metagenomic sequencing and ensures that complete functional pathways are contained within individual bacterium. In addition to improved accuracy, this method also has the capacity to improve sensitivity for functional analysis, allowing detection of functions that, although not prevalent, may represent fundamental differences between study cohorts.

Although extensive characterization of pathogens and model organisms has dominated the past 100 years of microbiology research, study of human-health-associated commensal bacteria has lagged behind. Culturing, genome sequencing and isolate archiving, as reported here, will underpin substantially improved microbiome-based analysis of the human gastrointestinal tract, and potentially other sites^28^. Traditional microbiology methods can continue to enable access to the bacterial isolates that are sorely needed to perform experimental characterization and validation and improving our understanding of important human-associated microbial communities.

## Methods

### Bacterial culturing and purification

Bacterial culturing was performed using supplemented YCFA medium^29^ with or without ethanol pretreatment as described previously^17^. Briefly, sample processing and culturing took place under anaerobic conditions in a Whitley DG250 workstation at 37 °C using phosphate buffered saline and culture media incubated under anaerobic conditions for 24 h before use. Fecal samples were collected from 20 healthy adults (North America, *n* = 12; United Kingdom, *n* = 8) who had not taken antibiotics within the last six months. Samples were transported frozen and stored at −80 °C before culturing. Fecal samples were homogenized in reduced PBS (0.1 g stool per ml PBS), serially diluted and plated directly onto YCFA^29^ agar supplemented with 0.002 g ml^−1^ each of glucose, maltose and cellobiose in (13.5-cm diameter) Petri dishes. Colonies were picked, restreaked to purity and identified using 16 S rRNA gene sequencing. Species were defined on the basis of a 16 S rRNA gene sequence identity threshold of >97.8%^30^. Isolates are available from the Wellcome Sanger Institute or the relevant public culture collection.

### Genome sequencing and annotation

Genomic DNA was extracted from pelleted cells using a phenol–chloroform method described previously^31^. DNA was prepared and sequenced using the Illumina Hi-Seq platform with library fragment sizes of 200–300 bp and a read length of 100 or 125 bp at the Wellcome Sanger Institute as previously described^32^. Annotated assemblies were produced using the pipeline described previously^33^. For each sample, sequence reads were used to create multiple assemblies using Velvet v1.2 (ref. ^34^) and VelvetOptimiser v2.2.5 (https://github.com/tseemann/VelvetOptimiser). An assembly improvement step was applied to the assembly with the best N50, and contigs were scaffolded using SSPACE^35^ and sequence gaps filled using GapFiller^36^. Automated annotation was performed using PROKKA v1.11 (ref. ^37^). Genomes with less than 400 contigs, a genome size less than 8 Mb and the presence of 16 s rRNA sequences with greater than 97.5% homology were considered pure and included in further analysis. All genomes within our collection are publicly available through the EBI European Nucleotide Archive under project accessions ERP105624 and ERP012217 (Supplementary Table 1). Public samples were included when the isolation source within the NCBI was fecal material or gastrointestinal-tract associated, and sequences were derived from pure isolates. All genomes were screened for quality as described for internal genomes, with only those passing these criteria included for further analysis.

### Phylogenetic analysis

The phylogenetic analysis was conducted by extracting amino acid sequence of 40 universal core marker genes^38,39^ from each genome in the bacterial collection using SpecI^21^. The protein sequences were concatenated and aligned with MAFFT v. 7.20 (ref. ^40^), and maximum-likelihood trees were constructed using RAxML v. 8.2.8 (ref. ^41^) with the standard LG model and 100 rapid bootstrap replicates. Trees were visualized using FastTree^42^ followed by iTOL^43^.

### De novo metagenomic analysis

For the metagenomic analyses, we first extracted 13,490 metagenomic sequencing runs from human gut samples available in the European Nucleotide Archive (Supplementary Table 4). To evaluate the efficiency of de novo assembly approaches, raw reads were assembled using metaSPAdes v3.10.0 (ref. ^44^) and subsequently binned with MetaBAT 2 (v2.12.1)^45^, with a minimum contig length threshold of 2,000 bp. Sequencing coverage and read base usage was inferred by mapping the raw reads back to the assemblies or bins using BWA v0.7.16 (ref. ^46^) and then retrieving the percentage of mapped read bases with SAMtools v1.5 (ref. ^46^) and the jgi_summarize_bam_contig_depths function from MetaBAT 2 (ref. ^45^). Ribosomal RNAs (rRNAs) were detected with INFERNAL v1.1.2 (ref. ^47^) using the Rfam covariance models of the bacterial 5 S, 16 S and 23 S rRNAs. Total alignment length was inferred by the sum of all nonoverlapping hits. Each gene was considered present if more than 80% of the sequence was contained in the MAG. Transfer RNAs (tRNAs) were identified with tRNAscan-SE v2.0 (ref. ^48^) using the bacterial tRNA model and default parameters. Bins with >90% completeness and < 5% contamination, estimated by CheckM^12^ were further analyzed against the Human Microbiome Project (HMP), the HBC genomes and complete HGG.

### Genome comparison

The complete HGG collection, the HBC-derived genomes and the HMP genomes were used for comparison to the MAGs. The complete HMP set, as well as a set of human gut-specific references retrieved from the HMP Project Catalog (https://www.hmpdacc.org/hmp/catalog/grid.php?dataset=genomic), were analyzed. For each database, Mash v2.0 (ref. ^49^) was used to convert all reference genomes into a MinHash sketch (mash sketch) with default settings. Then, the Mash distance between the MAGs and each set of references was calculated to find the best match (i.e., the genome with the lowest Mash distance). Each MAG and its closest relative among the reference set were aligned with dnadiff v1.3 from MUMmer 3.23 (ref. ^50^) to compare each pair in terms of percentage of aligned bases and ANI. MAGs that aligned above 75% of their total length with an ANI above 95% were considered a positive match. To further benchmark the assignment performance, we subsampled the reference genomes from each database at increments of 100 genomes and created MinHash sketches with a sketch size of 100,000 (*mash sketch -s 100,000*). We then assessed the number of MAGs that matched each subsampled set with a Mash distance below 0.05 (ANI > 95%). Data was visualized using the UpSet R package^51^.

### Lowest common ancestor metagenomic analysis

Lowest common ancestor analysis was performed using a custom generated Kraken database containing all genomes within the HBC. Metagenomic samples were filtered by quality using Trimmomatic 0.35 (ref. ^52^) and human contaminating reads filtered by mapping to the Human reference genome (hg19) with bowtie2 (ref. ^53^), with samples containing less than one million reads after filtering being discarded. Filtered sequences were classified at the genus and species levels using lowest common ancestor analysis as previously described^54^.

### Functional genomic analysis

To identify protein domains in a genome, we performed RPS-BLAST using COG database (accessed November 2017)^25^. All protein domains were classified in different functional categories using the COG database^25^ and were used to perform discriminant analysis of principle components (DAPC)^55^ implemented in the R package Adegenet v2.0.1 (ref. ^56^). Domain and functional enrichment analysis was calculated using one-sided Fisher’s exact test with *P* value adjusted by Hochberg method in R v. 3.2.2.

### Reporting Summary

Further information on research design is available in the Nature Research Reporting Summary linked to this article.

## Online content

Any methods, additional references, Nature Research reporting summaries, source data, statements of data availability and associated accession codes are available at 10.1038/s41587-018-0009-7.

## Supplementary Information

### Integrated supplementary information

Supplementary Figure 1 Counts of species and genome sequences within the HGG. Counts of species and genome sequences for each for Actinobacteria (*n* = 129 genomes, 55 species), Bacteroidetes (*n* = 231 genomes, 69 species), Firmicutes (*n* = 772 genomes, 339 species), Fusobacteria (*n* = 26 genomes, 9 species), Proteobacteria (*n* = 194 genomes, 56 species) and Synergistetes (*n* = 2 genomes, 2 species).

Supplementary Figure 2 Phylogenetic diversity of the human gastrointestinal microbiota genome collection. Maximum likelihood tree, including species names, generated using the 40 universal core genes from the 737 HBC genomes (green outer circle) and the 617 high-quality public genomes derived from human gastrointestinal tract samples, which together make up the HGG. Branch color distinguishes bacterial phyla belonging to Actinobacteria (gold), Bacteroidetes (green), Firmicutes (blue), Fusobacteria (brown), Synergistetes (black) and Proteobacteria (red) shown.

### Supplementary information

Supplementary Text and Figures Supplementary Figures 1 and 2

Reporting Summary

Supplementary Table 1 Species, ENA references, taxonomy and source for bacterial isolates within the HGG collection.

Supplementary Table 2 Metagenomic samples included in analysis.

Supplementary Table 3 39,913 metagenome-assembled genomes and associated quality metrics. Sequences are available for download from ftp://ftp.ebi.ac.uk/pub/databases/metagenomics/hgg_mags.tar.gz (32.6GB).

Supplementary Table 4 Mobile elements included in reference-based metagenomic analysis.

Supplementary Table 5 List of species present at levels greater than 0.01% within any two samples analyzed.

Supplementary Table 6 Core functions identified in every bacteria within the collection.

Supplementary Table 7 Functions overrepresented in each phylum relative to all functions present within the HGG (*q* < 0.001).

## Data Availability

Sequence data is deposited in the ENA under project numbers ERP105624 and ERP012217. Bacterial isolates have been deposited at the Leibniz Institute DSMZ-German Collection of Microorganims and Cell Cultures (http://www.dsmz.de), the CCUG-Culture Collection, University of Gothenburg, Sweden (http://www.ccug.se), the Belgian Co-ordinated Collection of Microorganisms hosted by the Laboratory of Microbiology (BCCM/LMG) at Ghent University (http://bccm.belspo.be/) and at the Japan Collection of Microorganisms (JCM; http://jcm.brc.riken.jp/en/). Culture collection identifiers and ENA accession numbers for each genome are provided in Supplementary Table 1. Metagenome-assembled genomes are available from ftp://ftp.ebi.ac.uk/pub/databases/metagenomics/hgg_mags.tar.gz.
