# Supplementary figures and images for: A human gut bacterial genome and culture collection for improved metagenomic analyses
Source: Nat Biotechnol. 2019 Feb 4;37(2):186–92. doi: 10.1038/s41587-018-0009-7 (PMC6785715; doi:10.1038/s41587-018-0009-7)

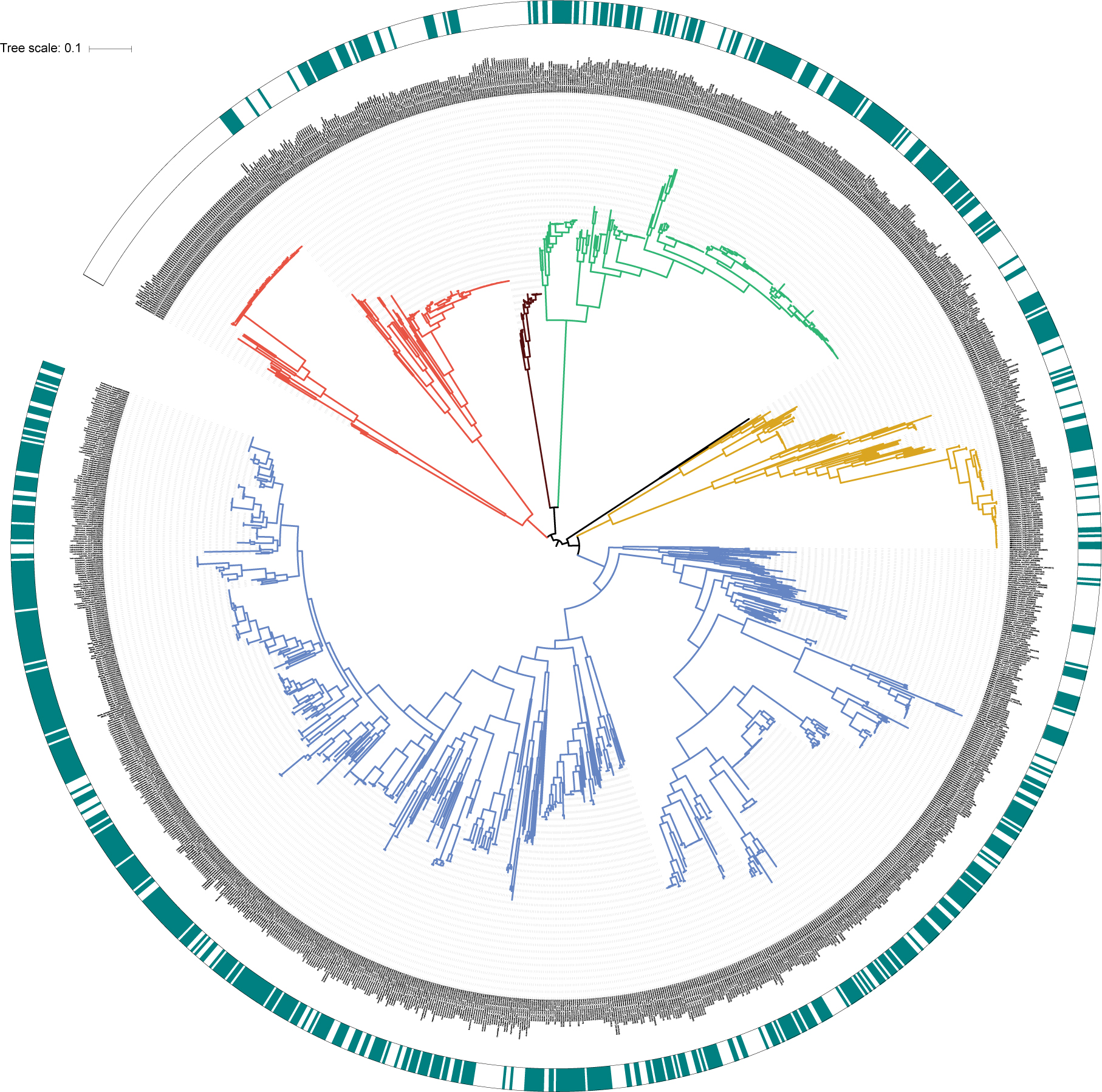

Supplement: Counts of species and genome sequences within the HGG. — Counts of species and genome sequences for each for Actinobacteria (n = 129 genomes, 55 species), Bacteroidetes (n = 231 genomes, 69 species), Firmicutes (n = 772 genomes, 339 species), Fusobacteria (n = 26 genomes, 9 species), Proteobacteria (n = 194 genomes, 56 species) and Synergistetes (n = 2 genomes, 2 species). [file 41587_2018_9_Fig6_ESM.jpg]

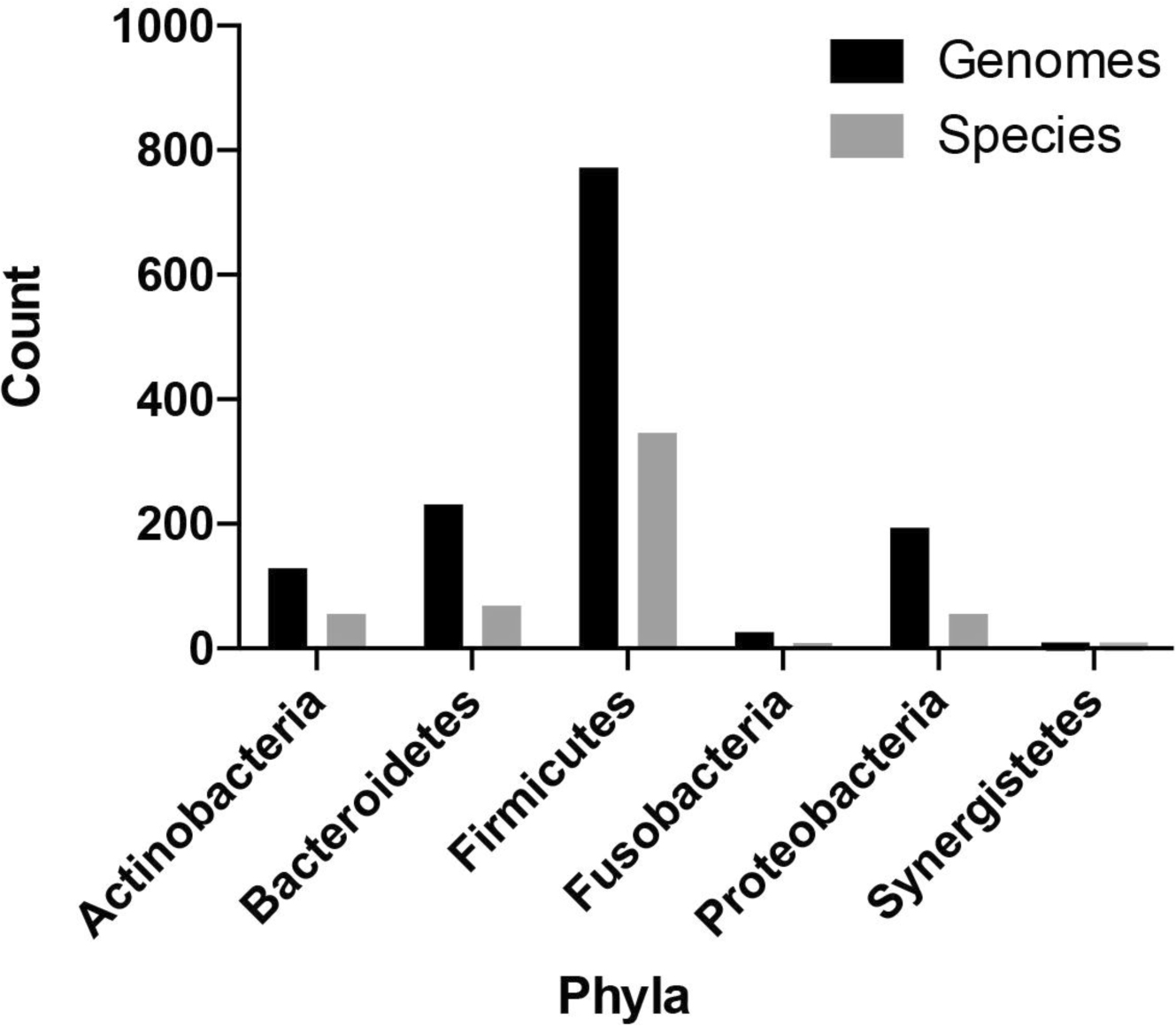

Supplement: Phylogenetic diversity of the human gastrointestinal microbiota genome collection. — Maximum likelihood tree, including species names, generated using the 40 universal core genes from the 737 HBC genomes (green outer circle) and the 617 high-quality public genomes derived from human gastrointestinal tract samples, which together make up the HGG. Branch color distinguishes bacterial phyla belonging to Actinobacteria (gold), Bacteroidetes (green), Firmicutes (blue), Fusobacteria (brown), Synergistetes (black) and Proteobacteria (red) shown. [file 41587_2018_9_Fig7_ESM.jpg]
