# Supplementary material for: A human gut bacterial genome and culture collection for improved metagenomic analyses
Source: Nat Biotechnol. 2019 Feb 4;37(2):186–92. doi: 10.1038/s41587-018-0009-7 (PMC6785715; doi:10.1038/s41587-018-0009-7)
Supplement: Supplementary file 3 — Supplementary Figures 1 and 2 [file 41587_2018_9_MOESM1_ESM.pdf]

nature biotechnology
SUPPLEMENTARY INFORMATION
https://doi.org/10.1038/s41587-018-0009-7

In the format provided by the authors and unedited.

A human gut bacterial genome and culture collection for improved metagenomic analyses

Samuel C. Forster[1,2,3,8*], Nitin Kumar[1,8], Blessing O. Anonye[1,7], Alexandre Almeida[4,5], Elisa Viciani[1], Mark D. Stares[1], Matthew Dunn[1], Tapoka T. Mkandawire[1], Ana Zhu[1], Yan Shao[1], Lindsay J. Pike[1], Thomas Louie[6], Hilary P. Browne[1], Alex L. Mitchell[4], B. Anne Neville[1], Robert D. Finn[4] and Trevor D. Lawley[1*]

[1]Host-Microbiota Interactions Laboratory, Wellcome Sanger Institute, Wellcome Genome Campus, Hinxton, UK. [2]Centre for Innate Immunity and Infectious Diseases, Hudson Institute of Medical Research, Clayton, Victoria, Australia. [3]Department of Molecular and Translational Sciences, Monash University, Clayton, Victoria, Australia. [4]European Molecular Biology Laboratory, European Bioinformatics Institute, Wellcome Genome Campus, Hinxton, UK. [5]Bacterial Genomics and Evolution Laboratory, Wellcome Sanger Institute, Wellcome Genome Campus, Hinxton, UK. [6]Department of Microbiology and Infectious Diseases, University of Calgary, Calgary, Alberta, Canada. [7]Present address: Microbiology and Infection Unit, Division of Biomedical Sciences, Warwick Medical School, University of Warwick, Coventry, UK. [8]These authors contributed equally: Samuel C. Forster, Nitin Kumar.
*e-mail: sf15@sanger.ac.uk; tl2@sanger.ac.uk

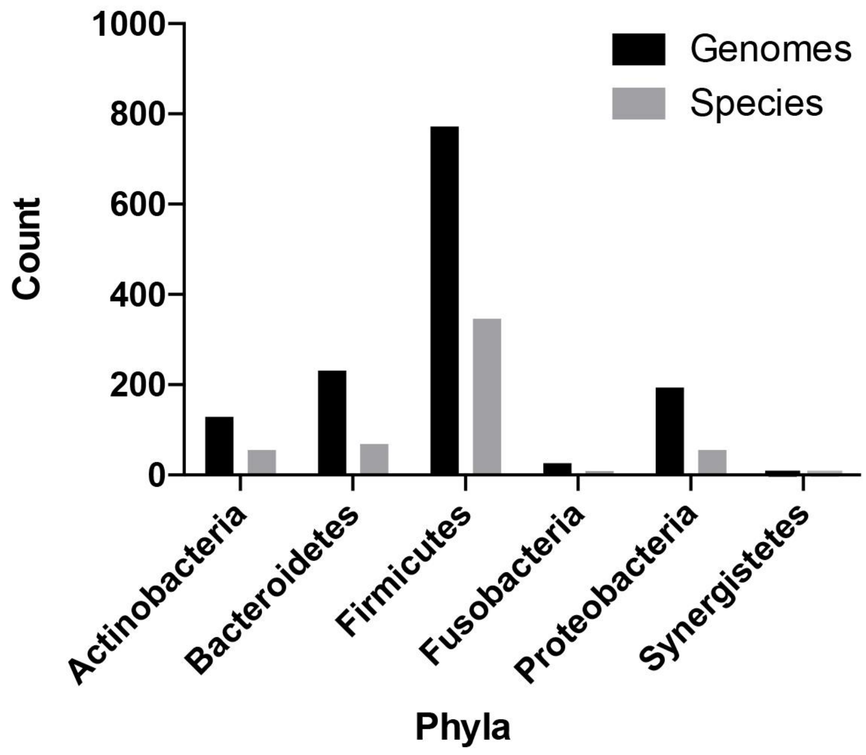

Supplementary Figure 1

Counts of species and genome sequences within the HGG

Counts of species and genome sequences for each for Actinobacteria (*n* = 129 genomes, 55 species), Bacteroidetes (*n* = 231 genomes, 69 species), Firmicutes (*n* = 772 genomes, 339 species), Fusobacteria (*n* = 26 genomes, 9 species), Proteobacteria (*n* = 194 genomes, 56 species) and Synergistetes (*n* = 2 genomes 2 species)

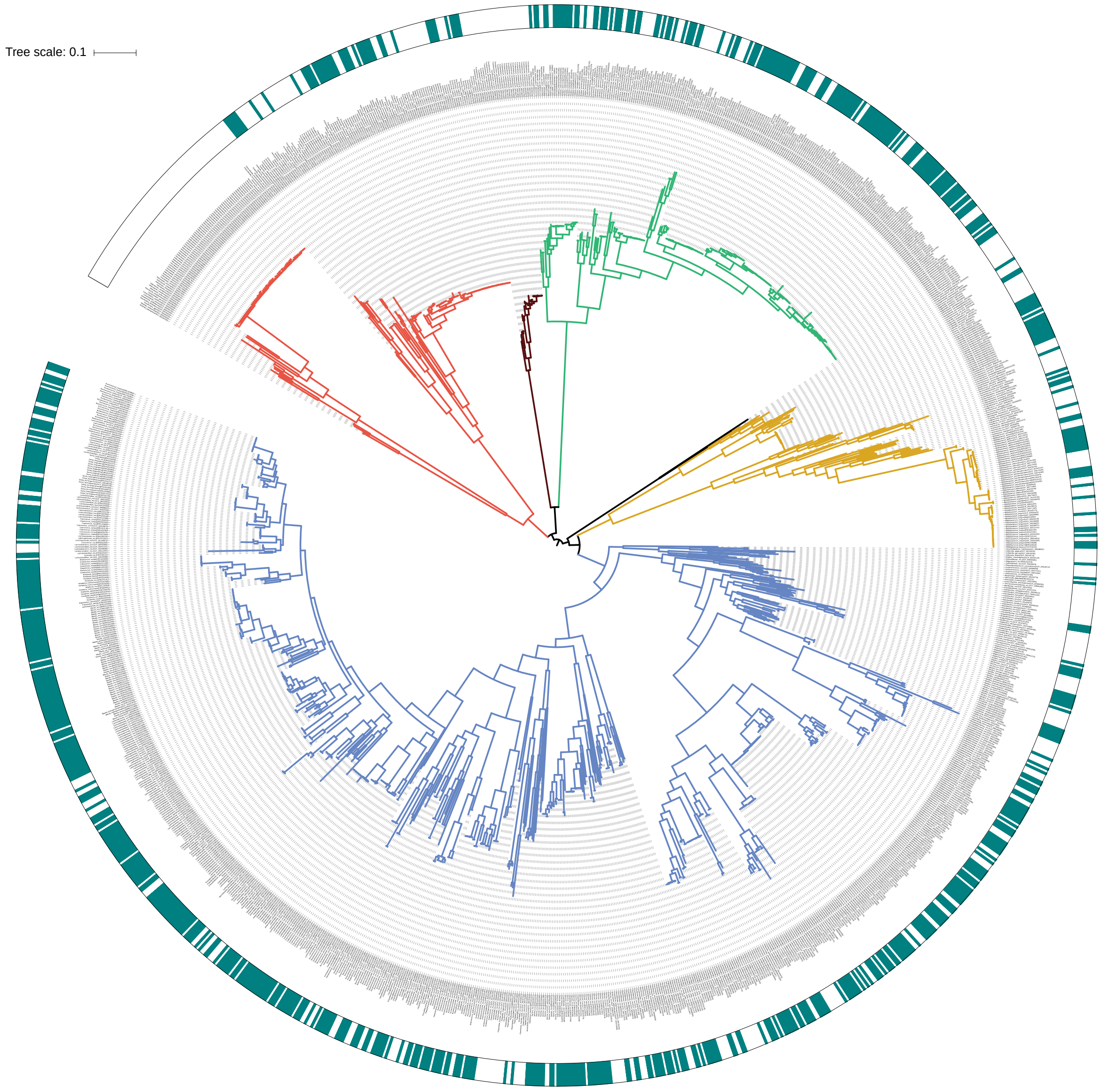

Supplementary Figure 2

Phylogenetic diversity of the human gastrointestinal microbiota genome collection.

Maximum likelihood tree, including species names, generated using the 40 universal core genes from the 737 HBC genomes (green outer circle) and the 617 high-quality public genomes derived from human gastrointestinal tract samples, which together make up the HGG. Branch color distinguishes bacterial phyla belonging to Actinobacteria (gold), Bacteroidetes (green), Firmicutes (blue), Fusobacteria (brown), Synergistetes (black) and Proteobacteria (red) shown.
