# Supplementary material for: A human gut bacterial genome and culture collection for improved metagenomic analyses
Source: Nat Biotechnol. 2019 Feb 4;37(2):186–92. doi: 10.1038/s41587-018-0009-7 (PMC6785715; doi:10.1038/s41587-018-0009-7)
Supplement: Supplementary file 4 — Reporting Summary [file 41587_2018_9_MOESM2_ESM.pdf]

nature research

Corresponding author(s): Samuel C Forster and Trevor D Lawley

Reporting Summary

Nature Research wishes to improve the reproducibility of the work that we publish. This form provides structure for consistency and transparency in reporting. For further information on Nature Research policies, see Authors & Referees and the Editorial Policy Checklist.

Statistical parameters

When statistical analyses are reported, confirm that the following items are present in the relevant location (e.g. figure legend, table legend, main text, or Methods section).

n/a	Confirmed	
☐	☒	The exact sample size (*n*) for each experimental group/condition, given as a discrete number and unit of measurement
☐	☒	An indication of whether measurements were taken from distinct samples or whether the same sample was measured repeatedly
☐	☒	The statistical test(s) used AND whether they are one- or two-sided<br>*Only common tests should be described solely by name; describe more complex techniques in the Methods section.*
☒	☐	A description of all covariates tested
☐	☒	A description of any assumptions or corrections, such as tests of normality and adjustment for multiple comparisons
☐	☒	A full description of the statistics including central tendency (e.g. means) or other basic estimates (e.g. regression coefficient) AND variation (e.g. standard deviation) or associated estimates of uncertainty (e.g. confidence intervals)
☐	☒	For null hypothesis testing, the test statistic (e.g. *F*, *t*, *r*) with confidence intervals, effect sizes, degrees of freedom and *P* value noted<br>*Give P values as exact values whenever suitable.*
☒	☐	For Bayesian analysis, information on the choice of priors and Markov chain Monte Carlo settings
☒	☐	For hierarchical and complex designs, identification of the appropriate level for tests and full reporting of outcomes
☒	☐	Estimates of effect sizes (e.g. Cohen's *d*, Pearson's *r*), indicating how they were calculated
☐	☒	Clearly defined error bars<br>*State explicitly what error bars represent (e.g. SD, SE, CI)*

Our web collection on statistics for biologists may be useful.

Software and code

Policy information about availability of computer code

Data collection	Illumina Sequencing data was collected using HCS 3.4.0.
Data analysis	Software used for data analysis: Velvet v1.2, VelvetOptimiser v2.2.5, SSPACE, GapFiller, PROKKA v1.11, MAFFT v. 7.20, RAxML v. 8.2.8, FastTree, iTOL, metaSPAdes v3.10.0, MetaBAT v2.12.1, BWA v0.7.16, samtools v1.5, MetaBAT 2 (jgi_summarize_bam_contig_depths), INFERNAL v1.1.2, tRNAscan-SE v2.0, CheckM, Mash v2.0, dnadiff v1.3, MUMmer v3.23, Kraken, Trimmomatic 0.35, bowtie2.<br>R v3.2.2: R Packages: UpSet R, Adegenet v2.0.1,

For manuscripts utilizing custom algorithms or software that are central to the research but not yet described in published literature, software must be made available to editors/reviewers upon request. We strongly encourage code deposition in a community repository (e.g. GitHub). See the Nature Research guidelines for submitting code & software for further information.

Data

Policy information about availability of data

All manuscripts must include a data availability statement. This statement should provide the following information, where applicable:

- Accession codes, unique identifiers, or web links for publicly available datasets
- A list of figures that have associated raw data
- A description of any restrictions on data availability

Sequence data is deposited in the ENA under project numbers ERP105624 and ERP012217. ENA accession numbers for each genome, culture collections details and strain identifiers are provided in Supplementary Table 1. Metagenome-assembled genomes are available from ftp://ftp.ebi.ac.uk/pub/databases/metagenomics/hgg_mags.tar.gz.

Field-specific reporting

Please select the best fit for your research. If you are not sure, read the appropriate sections before making your selection.

☒ Life sciences ☐ Behavioural & social sciences ☐ Ecological, evolutionary & environmental sciences

For a reference copy of the document with all sections, see nature.com/authors/policies/ReportingSummary-flat.pdf

Life sciences study design

All studies must disclose on these points even when the disclosure is negative.

Sample size	No sample size calculations were performed as sample size was limited by practical culturing capacity.
Data exclusions	No data was excluded from the analysis.
Replication	Isolates were cultured from all samples. Metagenomic analysis included all high-quality samples available in the European Nucleotide Archive so further replication was not possible.
Randomization	Isolates were cultured from all samples. Randomization is not relevant to this study.
Blinding	Isolates were cultured from all samples. Blinding is not relevant to this study.

Reporting for specific materials, systems and methods

Materials & experimental systems

n/a	Involved in the study	
☐	☒	Unique biological materials
☒	☐	Antibodies
☒	☐	Eukaryotic cell lines
☒	☐	Palaeontology
☒	☐	Animals and other organisms
☐	☒	Human research participants

Methods

n/a	Involved in the study	
☒	☐	ChIP-seq
☒	☐	Flow cytometry
☒	☐	MRI-based neuroimaging

Unique biological materials

Policy information about availability of materials

Obtaining unique materials	Bacterial isolates have been deposited at the Leibniz Institute DSMZ-German Collection of Microorganims and Cell Cultures (http://www.dsmz.de), the CCUG-Culture Collection, University of Gothenburg, Sweden (http://www.ccug.se), the Belgian Co-ordinated Collection of Micro-organisms hosted by the Laboratory of Microbiology (BCCM/LMG) at Ghent University (http://bccm.belspo.be/) or the Japan Collection of Microorganisms (JCM; http://jcm.brc.riken.jp/en/). Additional isolates are available upon request

Human research participants

Policy information about studies involving human research participants

Population characteristics	Samples were collected from healthy individuals between the ages of 25 and 55 with no reported antibiotic administration within the last 6 months. All samples were provided anonymously.
Recruitment	Healthy donors nominated to provide faecal samples for culturing in Canada and the UK. Donors with gastrointestinal disorders, chronic health conditions or with reported antibiotic administration within the last 6 months were excluded. Sampling of more diverse communities is likely to further increase the diversity of bacteria recovered.
